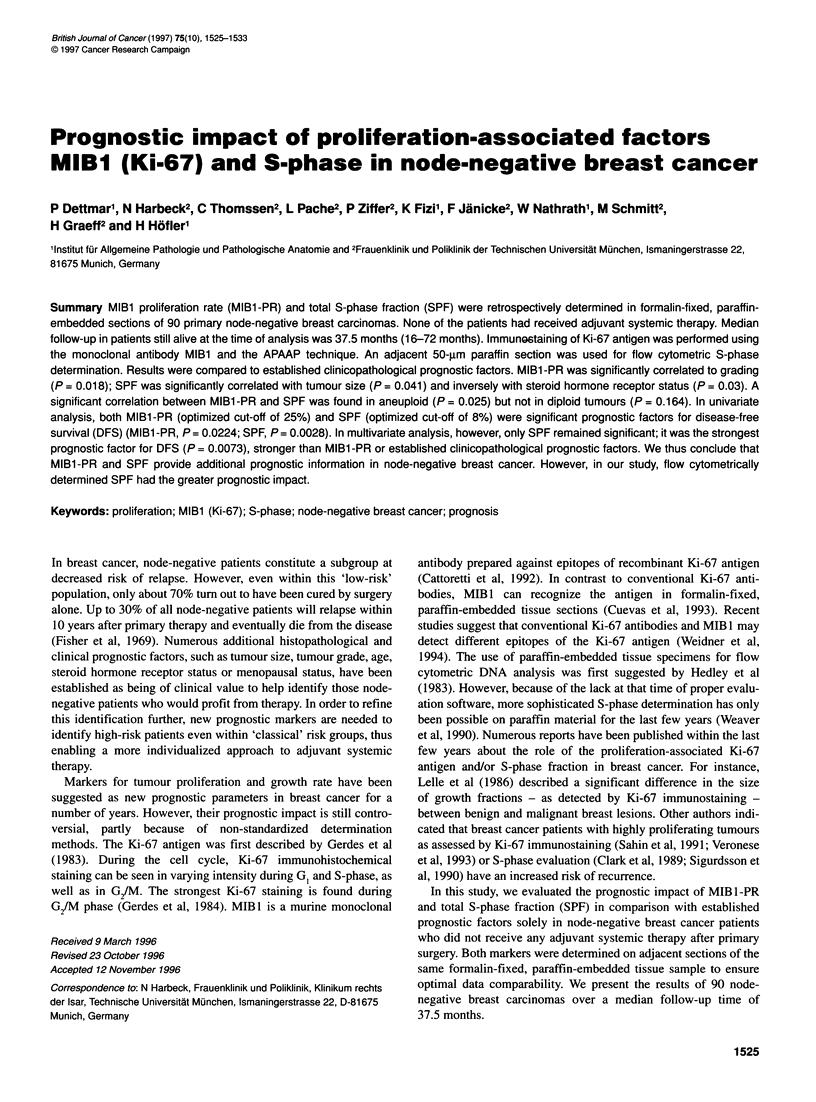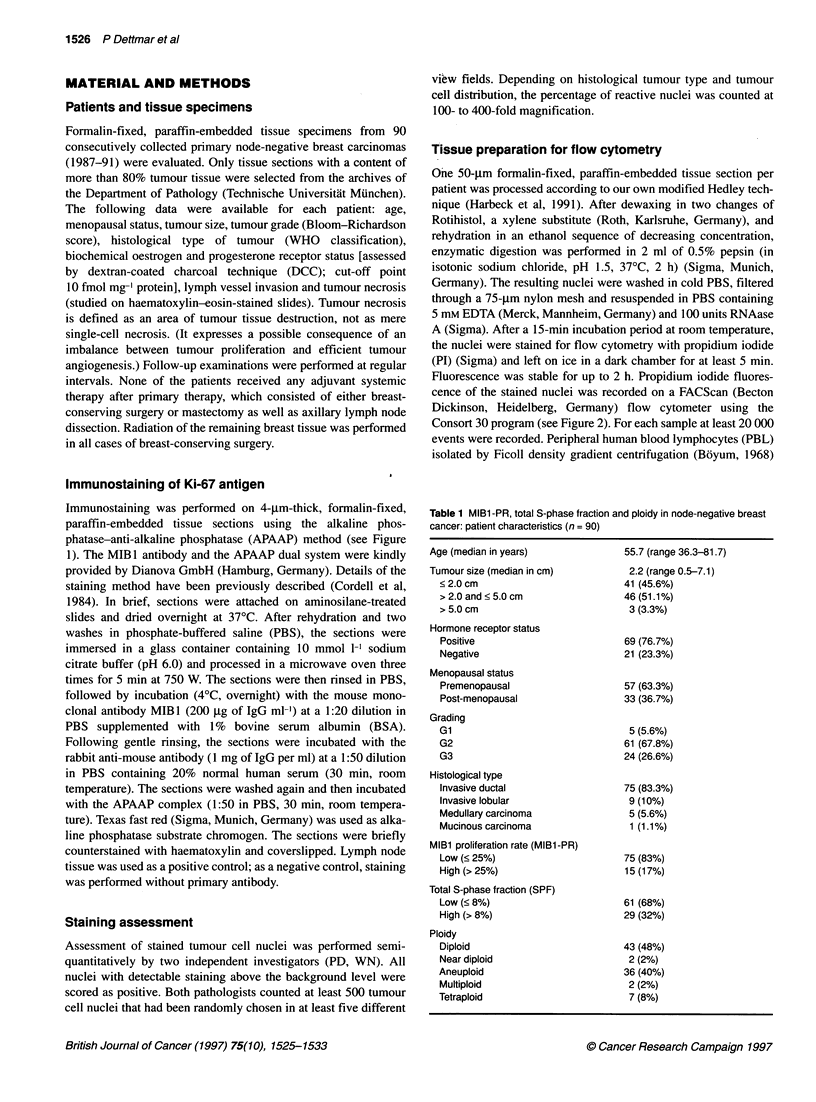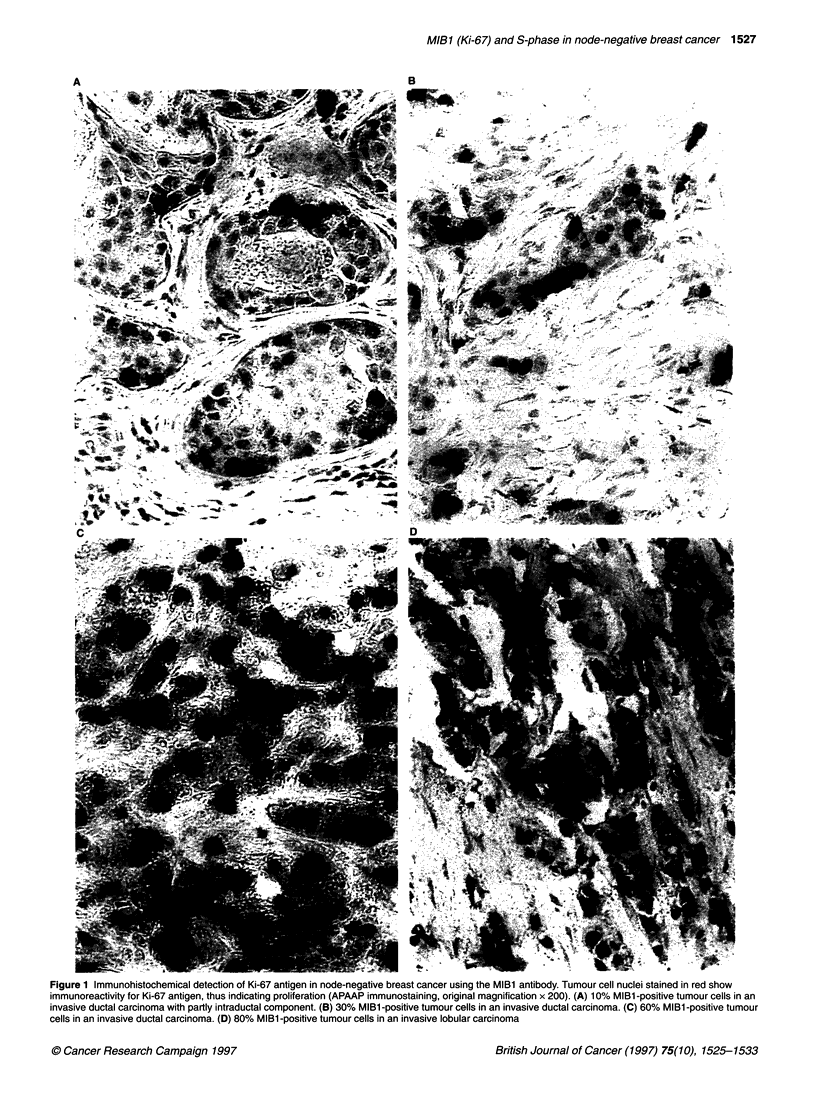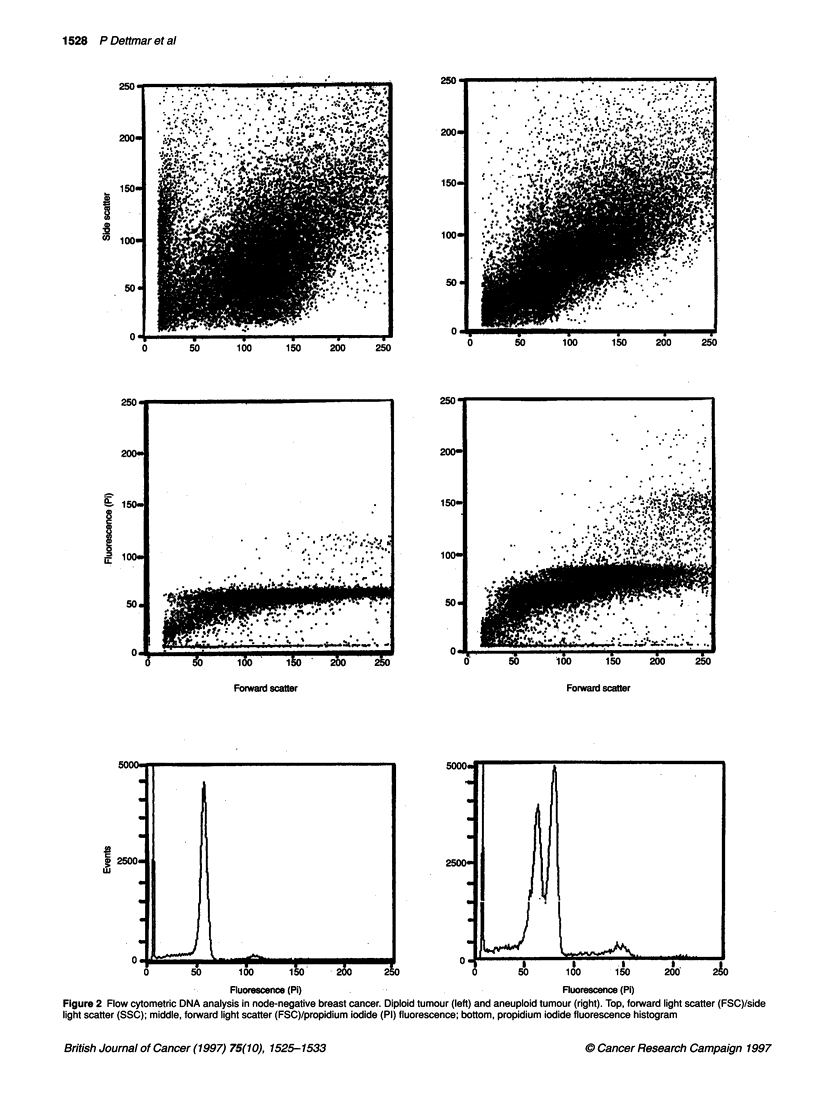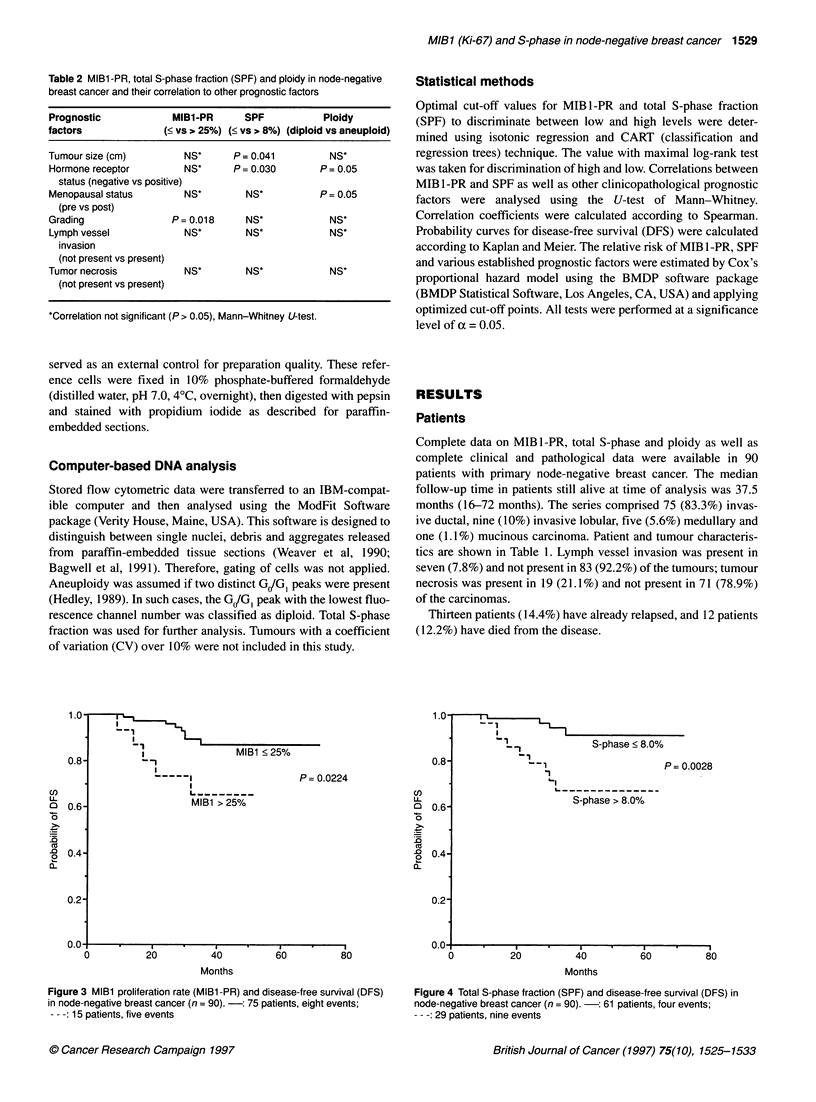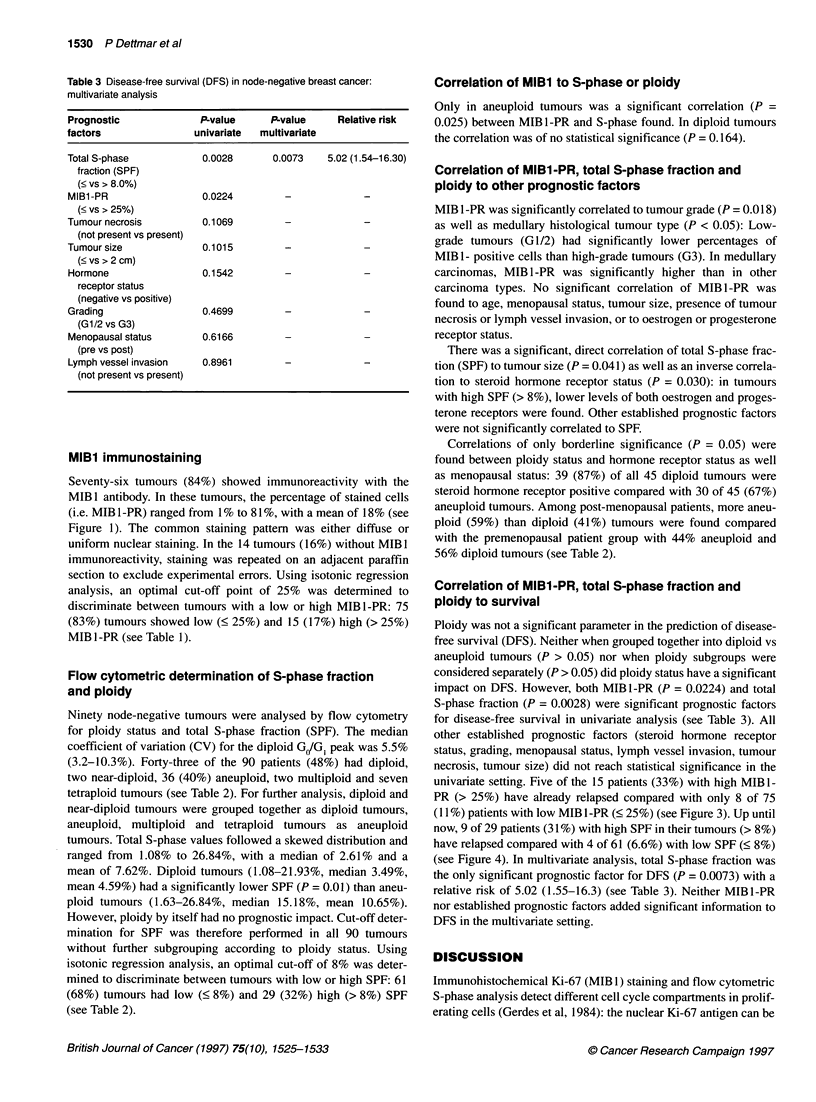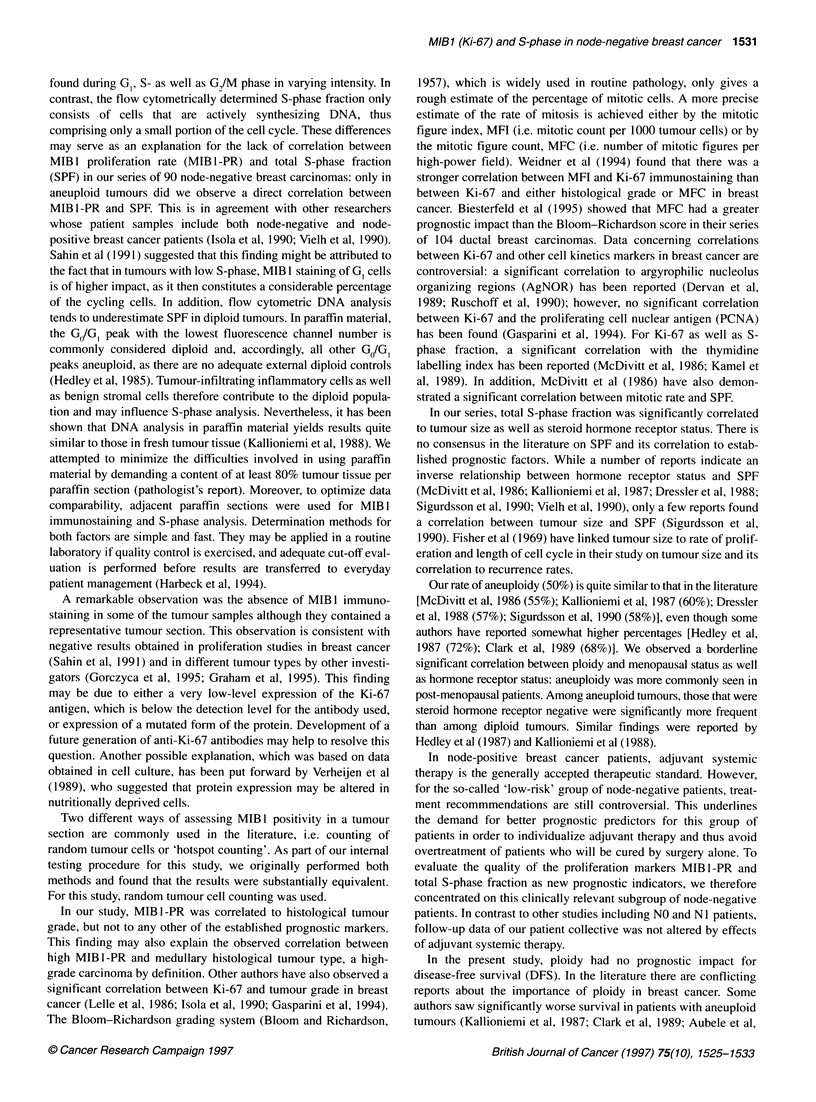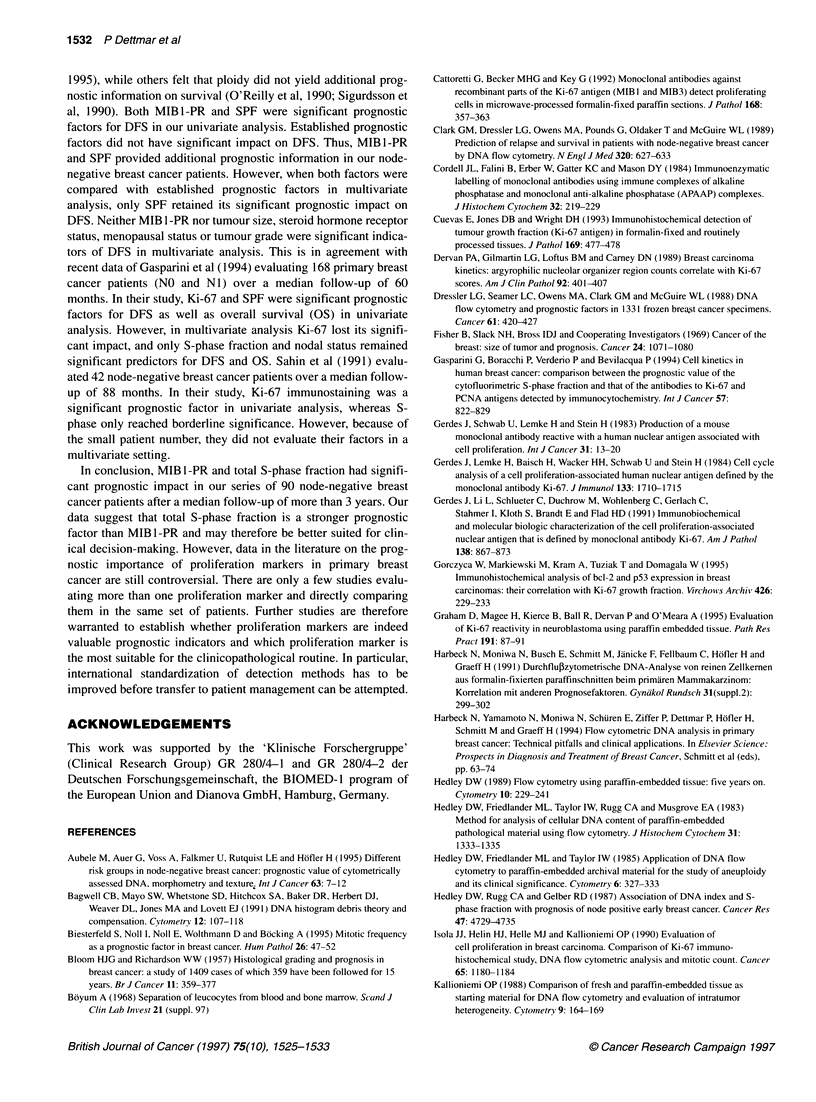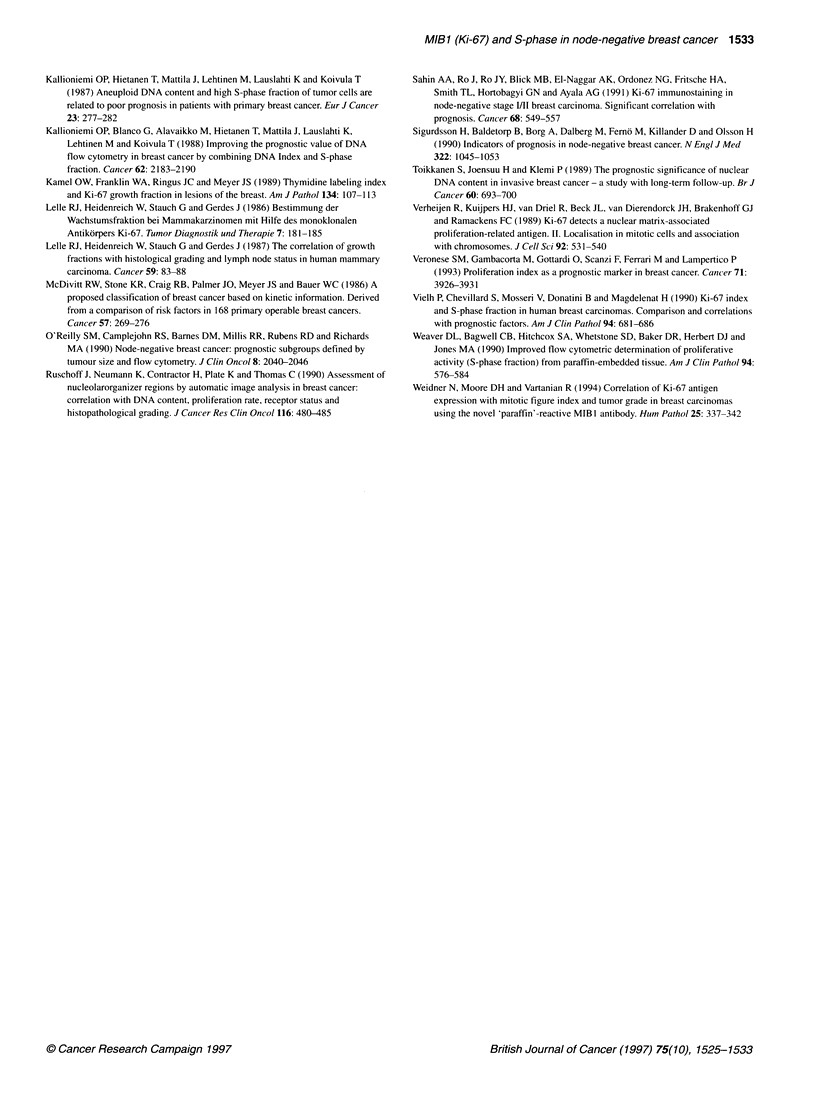# Prognostic impact of proliferation-associated factors MIB1 (Ki-67) and S-phase in node-negative breast cancer.

**DOI:** 10.1038/bjc.1997.261

**Published:** 1997

**Authors:** P. Dettmar, N. Harbeck, C. Thomssen, L. Pache, P. Ziffer, K. Fizi, F. JÃ¤nicke, W. Nathrath, M. Schmitt, H. Graeff, H. HÃ¶fler

**Affiliations:** Institut fÃ¼r Allgemeine Pathologie und Pathologische Anatomie, Munich,Germany.

## Abstract

**Images:**


British Journal of Cancer (1997) 75(10), 1525–1533
© 1997 Cancer Research Campaign

### Prognostic impact of proliferation-associated factors MIB1 (Ki-67) and S-phase in node-negative breast cancer

P Dettmar[1], N Harbeck[2], C Thomssen[2], L Pache[2], P Ziffer[2], K Fizi[1], F Jänicke[2], W Nathrath[1], M Schmitt[2], H Graeff[2] and H Höfler[1]

[1]Institut für Allgemeine Pathologie und Pathologische Anatomie and [2]Frauenklinik und Poliklinik der Technischen Universität München, Ismaningerstrasse 22, 81675 Munich, Germany

**Summary** MIB1 proliferation rate (MIB1-PR) and total S-phase fraction (SPF) were retrospectively determined in formalin-fixed, paraffin-embedded sections of 90 primary node-negative breast carcinomas. None of the patients had received adjuvant systemic therapy. Median follow-up in patients still alive at the time of analysis was 37.5 months (16–72 months). Immunostaining of Ki-67 antigen was performed using the monoclonal antibody MIB1 and the APAAP technique. An adjacent 50-μm paraffin section was used for flow cytometric S-phase determination. Results were compared to established clinicopathological prognostic factors. MIB1-PR was significantly correlated to grading ($P = 0.018$); SPF was significantly correlated with tumour size ($P = 0.041$) and inversely with steroid hormone receptor status ($P = 0.03$). A significant correlation between MIB1-PR and SPF was found in aneuploid ($P = 0.025$) but not in diploid tumours ($P = 0.164$). In univariate analysis, both MIB1-PR (optimized cut-off of 25%) and SPF (optimized cut-off of 8%) were significant prognostic factors for disease-free survival (DFS) (MIB1-PR, $P = 0.0224$; SPF, $P = 0.0028$). In multivariate analysis, however, only SPF remained significant; it was the strongest prognostic factor for DFS ($P = 0.0073$), stronger than MIB1-PR or established clinicopathological prognostic factors. We thus conclude that MIB1-PR and SPF provide additional prognostic information in node-negative breast cancer. However, in our study, flow cytometrically determined SPF had the greater prognostic impact.

**Keywords:** proliferation; MIB1 (Ki-67); S-phase; node-negative breast cancer; prognosis

In breast cancer, node-negative patients constitute a subgroup at decreased risk of relapse. However, even within this 'low-risk' population, only about 70% turn out to have been cured by surgery alone. Up to 30% of all node-negative patients will relapse within 10 years after primary therapy and eventually die from the disease (Fisher et al, 1969). Numerous additional histopathological and clinical prognostic factors, such as tumour size, tumour grade, age, steroid hormone receptor status or menopausal status, have been established as being of clinical value to help identify those node-negative patients who would profit from therapy. In order to refine this identification further, new prognostic markers are needed to identify high-risk patients even within 'classical' risk groups, thus enabling a more individualized approach to adjuvant systemic therapy.

Markers for tumour proliferation and growth rate have been suggested as new prognostic parameters in breast cancer for a number of years. However, their prognostic impact is still controversial, partly because of non-standardized determination methods. The Ki-67 antigen was first described by Gerdes et al (1983). During the cell cycle, Ki-67 immunohistochemical staining can be seen in varying intensity during $G_1$ and S-phase, as well as in $G_2$/M. The strongest Ki-67 staining is found during $G_2$/M phase (Gerdes et al, 1984). MIB1 is a murine monoclonal antibody prepared against epitopes of recombinant Ki-67 antigen (Cattoretti et al, 1992). In contrast to conventional Ki-67 antibodies, MIB1 can recognize the antigen in formalin-fixed, paraffin-embedded tissue sections (Cuevas et al, 1993). Recent studies suggest that conventional Ki-67 antibodies and MIB1 may detect different epitopes of the Ki-67 antigen (Weidner et al, 1994). The use of paraffin-embedded tissue specimens for flow cytometric DNA analysis was first suggested by Hedley et al (1983). However, because of the lack at that time of proper evaluation software, more sophisticated S-phase determination has only been possible on paraffin material for the last few years (Weaver et al, 1990). Numerous reports have been published within the last few years about the role of the proliferation-associated Ki-67 antigen and/or S-phase fraction in breast cancer. For instance, Lelle et al (1986) described a significant difference in the size of growth fractions – as detected by Ki-67 immunostaining – between benign and malignant breast lesions. Other authors indicated that breast cancer patients with highly proliferating tumours as assessed by Ki-67 immunostaining (Sahin et al, 1991; Veronese et al, 1993) or S-phase evaluation (Clark et al, 1989; Sigurdsson et al, 1990) have an increased risk of recurrence.

In this study, we evaluated the prognostic impact of MIB1-PR and total S-phase fraction (SPF) in comparison with established prognostic factors solely in node-negative breast cancer patients who did not receive any adjuvant systemic therapy after primary surgery. Both markers were determined on adjacent sections of the same formalin-fixed, paraffin-embedded tissue sample to ensure optimal data comparability. We present the results of 90 node-negative breast carcinomas over a median follow-up time of 37.5 months.

*Received 9 March 1996*
*Revised 23 October 1996*
*Accepted 12 November 1996*

*Correspondence to*: N Harbeck, Frauenklinik und Poliklinik, Klinikum rechts der Isar, Technische Universität München, Ismaningerstrasse 22, D-81675 Munich, Germany

### MATERIAL AND METHODS

### Patients and tissue specimens

Formalin-fixed, paraffin-embedded tissue specimens from 90 consecutively collected primary node-negative breast carcinomas (1987–91) were evaluated. Only tissue sections with a content of more than 80% tumour tissue were selected from the archives of the Department of Pathology (Technische Universität München). The following data were available for each patient: age, menopausal status, tumour size, tumour grade (Bloom–Richardson score), histological type of tumour (WHO classification), biochemical oestrogen and progesterone receptor status [assessed by dextran-coated charcoal technique (DCC); cut-off point 10 fmol $mg^{-1}$ protein], lymph vessel invasion and tumour necrosis (studied on haematoxylin–eosin-stained slides). Tumour necrosis is defined as an area of tumour tissue destruction, not as mere single-cell necrosis. (It expresses a possible consequence of an imbalance between tumour proliferation and efficient tumour angiogenesis.) Follow-up examinations were performed at regular intervals. None of the patients received any adjuvant systemic therapy after primary therapy, which consisted of either breast-conserving surgery or mastectomy as well as axillary lymph node dissection. Radiation of the remaining breast tissue was performed in all cases of breast-conserving surgery.

### Immunostaining of Ki-67 antigen

Immunostaining was performed on 4-μm-thick, formalin-fixed, paraffin-embedded tissue sections using the alkaline phosphatase–anti-alkaline phosphatase (APAAP) method (see Figure 1). The MIB1 antibody and the APAAP dual system were kindly provided by Dianova GmbH (Hamburg, Germany). Details of the staining method have been previously described (Cordell et al, 1984). In brief, sections were attached on aminosilane-treated slides and dried overnight at 37°C. After rehydration and two washes in phosphate-buffered saline (PBS), the sections were immersed in a glass container containing 10 mmol $l^{-1}$ sodium citrate buffer (pH 6.0) and processed in a microwave oven three times for 5 min at 750 W. The sections were then rinsed in PBS, followed by incubation (4°C, overnight) with the mouse monoclonal antibody MIB1 (200 μg of IgG $ml^{-1}$) at a 1:20 dilution in PBS supplemented with 1% bovine serum albumin (BSA). Following gentle rinsing, the sections were incubated with the rabbit anti-mouse antibody (1 mg of IgG per ml) at a 1:50 dilution in PBS containing 20% normal human serum (30 min, room temperature). The sections were washed again and then incubated with the APAAP complex (1:50 in PBS, 30 min, room temperature). Texas fast red (Sigma, Munich, Germany) was used as alkaline phosphatase substrate chromogen. The sections were briefly counterstained with haematoxylin and coverslipped. Lymph node tissue was used as a positive control; as a negative control, staining was performed without primary antibody.

### Staining assessment

Assessment of stained tumour cell nuclei was performed semi-quantitatively by two independent investigators (PD, WN). All nuclei with detectable staining above the background level were scored as positive. Both pathologists counted at least 500 tumour cell nuclei that had been randomly chosen in at least five different view fields. Depending on histological tumour type and tumour cell distribution, the percentage of reactive nuclei was counted at 100- to 400-fold magnification.

### Tissue preparation for flow cytometry

One 50-μm formalin-fixed, paraffin-embedded tissue section per patient was processed according to our own modified Hedley technique (Harbeck et al, 1991). After dewaxing in two changes of Rotihistol, a xylene substitute (Roth, Karlsruhe, Germany), and rehydration in an ethanol sequence of decreasing concentration, enzymatic digestion was performed in 2 ml of 0.5% pepsin (in isotonic sodium chloride, pH 1.5, 37°C, 2 h) (Sigma, Munich, Germany). The resulting nuclei were washed in cold PBS, filtered through a 75-μm nylon mesh and resuspended in PBS containing 5 mM EDTA (Merck, Mannheim, Germany) and 100 units RNAase A (Sigma). After a 15-min incubation period at room temperature, the nuclei were stained for flow cytometry with propidium iodide (PI) (Sigma) and left on ice in a dark chamber for at least 5 min. Fluorescence was stable for up to 2 h. Propidium iodide fluorescence of the stained nuclei was recorded on a FACScan (Becton Dickinson, Heidelberg, Germany) flow cytometer using the Consort 30 program (see Figure 2). For each sample at least 20 000 events were recorded. Peripheral human blood lymphocytes (PBL) isolated by Ficoll density gradient centrifugation (Böyum, 1968)

**Table 1** MIB1-PR, total S-phase fraction and ploidy in node-negative breast cancer: patient characteristics (*n* = 90)

| | |
|---|---|
| Age (median in years) | 55.7 (range 36.3–81.7) |
| Tumour size (median in cm) | 2.2 (range 0.5–7.1) |
| ≤ 2.0 cm | 41 (45.6%) |
| > 2.0 and ≤ 5.0 cm | 46 (51.1%) |
| > 5.0 cm | 3 (3.3%) |
| Hormone receptor status | |
| Positive | 69 (76.7%) |
| Negative | 21 (23.3%) |
| Menopausal status | |
| Premenopausal | 57 (63.3%) |
| Post-menopausal | 33 (36.7%) |
| Grading | |
| G1 | 5 (5.6%) |
| G2 | 61 (67.8%) |
| G3 | 24 (26.6%) |
| Histological type | |
| Invasive ductal | 75 (83.3%) |
| Invasive lobular | 9 (10%) |
| Medullary carcinoma | 5 (5.6%) |
| Mucinous carcinoma | 1 (1.1%) |
| MIB1 proliferation rate (MIB1-PR) | |
| Low (≤ 25%) | 75 (83%) |
| High (> 25%) | 15 (17%) |
| Total S-phase fraction (SPF) | |
| Low (≤ 8%) | 61 (68%) |
| High (> 8%) | 29 (32%) |
| Ploidy | |
| Diploid | 43 (48%) |
| Near diploid | 2 (2%) |
| Aneuploid | 36 (40%) |
| Multiploid | 2 (2%) |
| Tetraploid | 7 (8%) |

© Cancer Research Campaign 1997

**Figure 1** Immunohistochemical detection of Ki-67 antigen in node-negative breast cancer using the MIB1 antibody. Tumour cell nuclei stained in red show immunoreactivity for Ki-67 antigen, thus indicating proliferation (APAAP immunostaining, original magnification × 200). (**A**) 10% MIB1-positive tumour cells in an invasive ductal carcinoma with partly intraductal component. (**B**) 30% MIB1-positive tumour cells in an invasive ductal carcinoma. (**C**) 60% MIB1-positive tumour cells in an invasive ductal carcinoma. (**D**) 80% MIB1-positive tumour cells in an invasive lobular carcinoma

© Cancer Research Campaign 1997

**Figure 2** Flow cytometric DNA analysis in node-negative breast cancer. Diploid tumour (left) and aneuploid tumour (right). Top, forward light scatter (FSC)/side light scatter (SSC); middle, forward light scatter (FSC)/propidium iodide (PI) fluorescence; bottom, propidium iodide fluorescence histogram

 *© Cancer Research Campaign 1997*

**Table 2** MIB1-PR, total S-phase fraction (SPF) and ploidy in node-negative breast cancer and their correlation to other prognostic factors

| Prognostic factors | MIB1-PR (≤ vs > 25%) | SPF (≤ vs > 8%) | Ploidy (diploid vs aneuploid) |
|---|---|---|---|
| Tumour size (cm) | NS* | $P = 0.041$ | NS* |
| Hormone receptor status (negative vs positive) | NS* | $P = 0.030$ | $P = 0.05$ |
| Menopausal status (pre vs post) | NS* | NS* | $P = 0.05$ |
| Grading | $P = 0.018$ | NS* | NS* |
| Lymph vessel invasion (not present vs present) | NS* | NS* | NS* |
| Tumor necrosis (not present vs present) | NS* | NS* | NS* |

*Correlation not significant ($P > 0.05$), Mann–Whitney *U*-test.

served as an external control for preparation quality. These reference cells were fixed in 10% phosphate-buffered formaldehyde (distilled water, pH 7.0, 4°C, overnight), then digested with pepsin and stained with propidium iodide as described for paraffin-embedded sections.

### Computer-based DNA analysis

Stored flow cytometric data were transferred to an IBM-compatible computer and then analysed using the ModFit Software package (Verity House, Maine, USA). This software is designed to distinguish between single nuclei, debris and aggregates released from paraffin-embedded tissue sections (Weaver et al, 1990; Bagwell et al, 1991). Therefore, gating of cells was not applied. Aneuploidy was assumed if two distinct $G_0/G_1$ peaks were present (Hedley, 1989). In such cases, the $G_0/G_1$ peak with the lowest fluorescence channel number was classified as diploid. Total S-phase fraction was used for further analysis. Tumours with a coefficient of variation (CV) over 10% were not included in this study.

### Statistical methods

Optimal cut-off values for MIB1-PR and total S-phase fraction (SPF) to discriminate between low and high levels were determined using isotonic regression and CART (classification and regression trees) technique. The value with maximal log-rank test was taken for discrimination of high and low. Correlations between MIB1-PR and SPF as well as other clinicopathological prognostic factors were analysed using the *U*-test of Mann–Whitney. Correlation coefficients were calculated according to Spearman. Probability curves for disease-free survival (DFS) were calculated according to Kaplan and Meier. The relative risk of MIB1-PR, SPF and various established prognostic factors were estimated by Cox's proportional hazard model using the BMDP software package (BMDP Statistical Software, Los Angeles, CA, USA) and applying optimized cut-off points. All tests were performed at a significance level of $\alpha = 0.05$.

### RESULTS

### Patients

Complete data on MIB1-PR, total S-phase and ploidy as well as complete clinical and pathological data were available in 90 patients with primary node-negative breast cancer. The median follow-up time in patients still alive at time of analysis was 37.5 months (16–72 months). The series comprised 75 (83.3%) invasive ductal, nine (10%) invasive lobular, five (5.6%) medullary and one (1.1%) mucinous carcinoma. Patient and tumour characteristics are shown in Table 1. Lymph vessel invasion was present in seven (7.8%) and not present in 83 (92.2%) of the tumours; tumour necrosis was present in 19 (21.1%) and not present in 71 (78.9%) of the carcinomas.

Thirteen patients (14.4%) have already relapsed, and 12 patients (12.2%) have died from the disease.

**Figure 3** MIB1 proliferation rate (MIB1-PR) and disease-free survival (DFS) in node-negative breast cancer ($n = 90$). ——: 75 patients, eight events; - - -: 15 patients, five events

**Figure 4** Total S-phase fraction (SPF) and disease-free survival (DFS) in node-negative breast cancer ($n = 90$). ——: 61 patients, four events; - - -: 29 patients, nine events

© Cancer Research Campaign 1997 

Table 3 Disease-free survival (DFS) in node-negative breast cancer: multivariate analysis

| Prognostic factors | P-value univariate | P-value multivariate | Relative risk |
|---|---|---|---|
| Total S-phase fraction (SPF) (≤ vs > 8.0%) | 0.0028 | 0.0073 | 5.02 (1.54–16.30) |
| MIB1-PR (≤ vs > 25%) | 0.0224 | – | – |
| Tumour necrosis (not present vs present) | 0.1069 | – | – |
| Tumour size (≤ vs > 2 cm) | 0.1015 | – | – |
| Hormone receptor status (negative vs positive) | 0.1542 | – | – |
| Grading (G1/2 vs G3) | 0.4699 | – | – |
| Menopausal status (pre vs post) | 0.6166 | – | – |
| Lymph vessel invasion (not present vs present) | 0.8961 | – | – |

### MIB1 immunostaining

Seventy-six tumours (84%) showed immunoreactivity with the MIB1 antibody. In these tumours, the percentage of stained cells (i.e. MIB1-PR) ranged from 1% to 81%, with a mean of 18% (see Figure 1). The common staining pattern was either diffuse or uniform nuclear staining. In the 14 tumours (16%) without MIB1 immunoreactivity, staining was repeated on an adjacent paraffin section to exclude experimental errors. Using isotonic regression analysis, an optimal cut-off point of 25% was determined to discriminate between tumours with a low or high MIB1-PR: 75 (83%) tumours showed low (≤ 25%) and 15 (17%) high (> 25%) MIB1-PR (see Table 1).

### Flow cytometric determination of S-phase fraction and ploidy

Ninety node-negative tumours were analysed by flow cytometry for ploidy status and total S-phase fraction (SPF). The median coefficient of variation (CV) for the diploid $G_0/G_1$ peak was 5.5% (3.2–10.3%). Forty-three of the 90 patients (48%) had diploid, two near-diploid, 36 (40%) aneuploid, two multiploid and seven tetraploid tumours (see Table 2). For further analysis, diploid and near-diploid tumours were grouped together as diploid tumours, aneuploid, multiploid and tetraploid tumours as aneuploid tumours. Total S-phase values followed a skewed distribution and ranged from 1.08% to 26.84%, with a median of 2.61% and a mean of 7.62%. Diploid tumours (1.08–21.93%, median 3.49%, mean 4.59%) had a significantly lower SPF ($P$ = 0.01) than aneuploid tumours (1.63–26.84%, median 15.18%, mean 10.65%). However, ploidy by itself had no prognostic impact. Cut-off determination for SPF was therefore performed in all 90 tumours without further subgrouping according to ploidy status. Using isotonic regression analysis, an optimal cut-off of 8% was determined to discriminate between tumours with low or high SPF: 61 (68%) tumours had low (≤ 8%) and 29 (32%) high (> 8%) SPF (see Table 2).

### Correlation of MIB1 to S-phase or ploidy

Only in aneuploid tumours was a significant correlation ($P$ = 0.025) between MIB1-PR and S-phase found. In diploid tumours the correlation was of no statistical significance ($P$ = 0.164).

### Correlation of MIB1-PR, total S-phase fraction and ploidy to other prognostic factors

MIB1-PR was significantly correlated to tumour grade ($P$ = 0.018) as well as medullary histological tumour type ($P$ < 0.05): Low-grade tumours (G1/2) had significantly lower percentages of MIB1- positive cells than high-grade tumours (G3). In medullary carcinomas, MIB1-PR was significantly higher than in other carcinoma types. No significant correlation of MIB1-PR was found to age, menopausal status, tumour size, presence of tumour necrosis or lymph vessel invasion, or to oestrogen or progesterone receptor status.

There was a significant, direct correlation of total S-phase fraction (SPF) to tumour size ($P$ = 0.041) as well as an inverse correlation to steroid hormone receptor status ($P$ = 0.030): in tumours with high SPF (> 8%), lower levels of both oestrogen and progesterone receptors were found. Other established prognostic factors were not significantly correlated to SPF.

Correlations of only borderline significance ($P$ = 0.05) were found between ploidy status and hormone receptor status as well as menopausal status: 39 (87%) of all 45 diploid tumours were steroid hormone receptor positive compared with 30 of 45 (67%) aneuploid tumours. Among post-menopausal patients, more aneuploid (59%) than diploid (41%) tumours were found compared with the premenopausal patient group with 44% aneuploid and 56% diploid tumours (see Table 2).

### Correlation of MIB1-PR, total S-phase fraction and ploidy to survival

Ploidy was not a significant parameter in the prediction of disease-free survival (DFS). Neither when grouped together into diploid vs aneuploid tumours ($P$ > 0.05) nor when ploidy subgroups were considered separately ($P$ > 0.05) did ploidy status have a significant impact on DFS. However, both MIB1-PR ($P$ = 0.0224) and total S-phase fraction ($P$ = 0.0028) were significant prognostic factors for disease-free survival in univariate analysis (see Table 3). All other established prognostic factors (steroid hormone receptor status, grading, menopausal status, lymph vessel invasion, tumour necrosis, tumour size) did not reach statistical significance in the univariate setting. Five of the 15 patients (33%) with high MIB1-PR (> 25%) have already relapsed compared with only 8 of 75 (11%) patients with low MIB1-PR (≤ 25%) (see Figure 3). Up until now, 9 of 29 patients (31%) with high SPF in their tumours (> 8%) have relapsed compared with 4 of 61 (6.6%) with low SPF (≤ 8%) (see Figure 4). In multivariate analysis, total S-phase fraction was the only significant prognostic factor for DFS ($P$ = 0.0073) with a relative risk of 5.02 (1.55–16.3) (see Table 3). Neither MIB1-PR nor established prognostic factors added significant information to DFS in the multivariate setting.

### DISCUSSION

Immunohistochemical Ki-67 (MIB1) staining and flow cytometric S-phase analysis detect different cell cycle compartments in proliferating cells (Gerdes et al, 1984): the nuclear Ki-67 antigen can be

© Cancer Research Campaign 1997

found during $G_1$, S- as well as $G_2$/M phase in varying intensity. In contrast, the flow cytometrically determined S-phase fraction only consists of cells that are actively synthesizing DNA, thus comprising only a small portion of the cell cycle. These differences may serve as an explanation for the lack of correlation between MIB1 proliferation rate (MIB1-PR) and total S-phase fraction (SPF) in our series of 90 node-negative breast carcinomas: only in aneuploid tumours did we observe a direct correlation between MIB1-PR and SPF. This is in agreement with other researchers whose patient samples include both node-negative and node-positive breast cancer patients (Isola et al, 1990; Vielh et al, 1990). Sahin et al (1991) suggested that this finding might be attributed to the fact that in tumours with low S-phase, MIB1 staining of $G_1$ cells is of higher impact, as it then constitutes a considerable percentage of the cycling cells. In addition, flow cytometric DNA analysis tends to underestimate SPF in diploid tumours. In paraffin material, the $G_0$/$G_1$ peak with the lowest fluorescence channel number is commonly considered diploid and, accordingly, all other $G_0$/$G_1$ peaks aneuploid, as there are no adequate external diploid controls (Hedley et al, 1985). Tumour-infiltrating inflammatory cells as well as benign stromal cells therefore contribute to the diploid population and may influence S-phase analysis. Nevertheless, it has been shown that DNA analysis in paraffin material yields results quite similar to those in fresh tumour tissue (Kallioniemi et al, 1988). We attempted to minimize the difficulties involved in using paraffin material by demanding a content of at least 80% tumour tissue per paraffin section (pathologist's report). Moreover, to optimize data comparability, adjacent paraffin sections were used for MIB1 immunostaining and S-phase analysis. Determination methods for both factors are simple and fast. They may be applied in a routine laboratory if quality control is exercised, and adequate cut-off evaluation is performed before results are transferred to everyday patient management (Harbeck et al, 1994).

A remarkable observation was the absence of MIB1 immunostaining in some of the tumour samples although they contained a representative tumour section. This observation is consistent with negative results obtained in proliferation studies in breast cancer (Sahin et al, 1991) and in different tumour types by other investigators (Gorczyca et al, 1995; Graham et al, 1995). This finding may be due to either a very low-level expression of the Ki-67 antigen, which is below the detection level for the antibody used, or expression of a mutated form of the protein. Development of a future generation of anti-Ki-67 antibodies may help to resolve this question. Another possible explanation, which was based on data obtained in cell culture, has been put forward by Verheijen et al (1989), who suggested that protein expression may be altered in nutritionally deprived cells.

Two different ways of assessing MIB1 positivity in a tumour section are commonly used in the literature, i.e. counting of random tumour cells or 'hotspot counting'. As part of our internal testing procedure for this study, we originally performed both methods and found that the results were substantially equivalent. For this study, random tumour cell counting was used.

In our study, MIB1-PR was correlated to histological tumour grade, but not to any other of the established prognostic markers. This finding may also explain the observed correlation between high MIB1-PR and medullary histological tumour type, a high-grade carcinoma by definition. Other authors have also observed a significant correlation between Ki-67 and tumour grade in breast cancer (Lelle et al, 1986; Isola et al, 1990; Gasparini et al, 1994). The Bloom–Richardson grading system (Bloom and Richardson, 1957), which is widely used in routine pathology, only gives a rough estimate of the percentage of mitotic cells. A more precise estimate of the rate of mitosis is achieved either by the mitotic figure index, MFI (i.e. mitotic count per 1000 tumour cells) or by the mitotic figure count, MFC (i.e. number of mitotic figures per high-power field). Weidner et al (1994) found that there was a stronger correlation between MFI and Ki-67 immunostaining than between Ki-67 and either histological grade or MFC in breast cancer. Biesterfeld et al (1995) showed that MFC had a greater prognostic impact than the Bloom–Richardson score in their series of 104 ductal breast carcinomas. Data concerning correlations between Ki-67 and other cell kinetics markers in breast cancer are controversial: a significant correlation to argyrophilic nucleolus organizing regions (AgNOR) has been reported (Dervan et al, 1989; Ruschoff et al, 1990); however, no significant correlation between Ki-67 and the proliferating cell nuclear antigen (PCNA) has been found (Gasparini et al, 1994). For Ki-67 as well as S-phase fraction, a significant correlation with the thymidine labelling index has been reported (McDivitt et al, 1986; Kamel et al, 1989). In addition, McDivitt et al (1986) have also demonstrated a significant correlation between mitotic rate and SPF.

In our series, total S-phase fraction was significantly correlated to tumour size as well as steroid hormone receptor status. There is no consensus in the literature on SPF and its correlation to established prognostic factors. While a number of reports indicate an inverse relationship between hormone receptor status and SPF (McDivitt et al, 1986; Kallioniemi et al, 1987; Dressler et al, 1988; Sigurdsson et al, 1990; Vielh et al, 1990), only a few reports found a correlation between tumour size and SPF (Sigurdsson et al, 1990). Fisher et al (1969) have linked tumour size to rate of proliferation and length of cell cycle in their study on tumour size and its correlation to recurrence rates.

Our rate of aneuploidy (50%) is quite similar to that in the literature [McDivitt et al, 1986 (55%); Kallioniemi et al, 1987 (60%); Dressler et al, 1988 (57%); Sigurdsson et al, 1990 (58%)], even though some authors have reported somewhat higher percentages [Hedley et al, 1987 (72%); Clark et al, 1989 (68%)]. We observed a borderline significant correlation between ploidy and menopausal status as well as hormone receptor status: aneuploidy was more commonly seen in post-menopausal patients. Among aneuploid tumours, those that were steroid hormone receptor negative were significantly more frequent than among diploid tumours. Similar findings were reported by Hedley et al (1987) and Kallioniemi et al (1988).

In node-positive breast cancer patients, adjuvant systemic therapy is the generally accepted therapeutic standard. However, for the so-called 'low-risk' group of node-negative patients, treatment recommmendations are still controversial. This underlines the demand for better prognostic predictors for this group of patients in order to individualize adjuvant therapy and thus avoid overtreatment of patients who will be cured by surgery alone. To evaluate the quality of the proliferation markers MIB1-PR and total S-phase fraction as new prognostic indicators, we therefore concentrated on this clinically relevant subgroup of node-negative patients. In contrast to other studies including N0 and N1 patients, follow-up data of our patient collective was not altered by effects of adjuvant systemic therapy.

In the present study, ploidy had no prognostic impact for disease-free survival (DFS). In the literature there are conflicting reports about the importance of ploidy in breast cancer. Some authors saw significantly worse survival in patients with aneuploid tumours (Kallioniemi et al, 1987; Clark et al, 1989; Aubele et al,

© Cancer Research Campaign 1997

1995), while others felt that ploidy did not yield additional prognostic information on survival (O'Reilly et al, 1990; Sigurdsson et al, 1990). Both MIB1-PR and SPF were significant prognostic factors for DFS in our univariate analysis. Established prognostic factors did not have significant impact on DFS. Thus, MIB1-PR and SPF provided additional prognostic information in our node-negative breast cancer patients. However, when both factors were compared with established prognostic factors in multivariate analysis, only SPF retained its significant prognostic impact on DFS. Neither MIB1-PR nor tumour size, steroid hormone receptor status, menopausal status or tumour grade were significant indicators of DFS in multivariate analysis. This is in agreement with recent data of Gasparini et al (1994) evaluating 168 primary breast cancer patients (N0 and N1) over a median follow-up of 60 months. In their study, Ki-67 and SPF were significant prognostic factors for DFS as well as overall survival (OS) in univariate analysis. However, in multivariate analysis Ki-67 lost its significant impact, and only S-phase fraction and nodal status remained significant predictors for DFS and OS. Sahin et al (1991) evaluated 42 node-negative breast cancer patients over a median follow-up of 88 months. In their study, Ki-67 immunostaining was a significant prognostic factor in univariate analysis, whereas S-phase only reached borderline significance. However, because of the small patient number, they did not evaluate their factors in a multivariate setting.

In conclusion, MIB1-PR and total S-phase fraction had significant prognostic impact in our series of 90 node-negative breast cancer patients after a median follow-up of more than 3 years. Our data suggest that total S-phase fraction is a stronger prognostic factor than MIB1-PR and may therefore be better suited for clinical decision-making. However, data in the literature on the prognostic importance of proliferation markers in primary breast cancer are still controversial. There are only a few studies evaluating more than one proliferation marker and directly comparing them in the same set of patients. Further studies are therefore warranted to establish whether proliferation markers are indeed valuable prognostic indicators and which proliferation marker is the most suitable for the clinicopathological routine. In particular, international standardization of detection methods has to be improved before transfer to patient management can be attempted.

### ACKNOWLEDGEMENTS

This work was supported by the 'Klinische Forschergruppe' (Clinical Research Group) GR 280/4–1 and GR 280/4–2 der Deutschen Forschungsgemeinschaft, the BIOMED-1 program of the European Union and Dianova GmbH, Hamburg, Germany.

### REFERENCES

Aubele M, Auer G, Voss A, Falkmer U, Rutquist LE and Höfler H (1995) Different risk groups in node-negative breast cancer: prognostic value of cytometrically assessed DNA, morphometry and texture. *Int J Cancer* **63**: 7–12

Bagwell CB, Mayo SW, Whetstone SD, Hitchcox SA, Baker DR, Herbert DJ, Weaver DL, Jones MA and Lovett EJ (1991) DNA histogram debris theory and compensation. *Cytometry* **12**: 107–118

Biesterfeld S, Noll I, Noll E, Wolthmann D and Böcking A (1995) Mitotic frequency as a prognostic factor in breast cancer. *Hum Pathol* **26**: 47–52

Bloom HJG and Richardson WW (1957) Histological grading and prognosis in breast cancer: a study of 1409 cases of which 359 have been followed for 15 years. *Br J Cancer* **11**: 359–377

Böyum A (1968) Separation of leucocytes from blood and bone marrow. *Scand J Clin Lab Invest* **21** (suppl. 97)

Cattoretti G, Becker MHG and Key G (1992) Monoclonal antibodies against recombinant parts of the Ki-67 antigen (MIB1 and MIB3) detect proliferating cells in microwave-processed formalin-fixed paraffin sections. *J Pathol* **168**: 357–363

Clark GM, Dressler LG, Owens MA, Pounds G, Oldaker T and McGuire WL (1989) Prediction of relapse and survival in patients with node-negative breast cancer by DNA flow cytometry. *N Engl J Med* **320**: 627–633

Cordell JL, Falini B, Erber W, Gatter KC and Mason DY (1984) Immunoenzymatic labelling of monoclonal antibodies using immune complexes of alkaline phosphatase and monoclonal anti-alkaline phosphatase (APAAP) complexes. *J Histochem Cytochem* **32**: 219–229

Cuevas E, Jones DB and Wright DH (1993) Immunohistochemical detection of tumour growth fraction (Ki-67 antigen) in formalin-fixed and routinely processed tissues. *J Pathol* **169**: 477–478

Dervan PA, Gilmartin LG, Loftus BM and Carney DN (1989) Breast carcinoma kinetics: argyrophilic nucleolar organizer region counts correlate with Ki-67 scores. *Am J Clin Pathol* **92**: 401–407

Dressler LG, Seamer LC, Owens MA, Clark GM and McGuire WL (1988) DNA flow cytometry and prognostic factors in 1331 frozen breast cancer specimens. *Cancer* **61**: 420–427

Fisher B, Slack NH, Bross IDJ and Cooperating Investigators (1969) Cancer of the breast: size of tumor and prognosis. *Cancer* **24**: 1071–1080

Gasparini G, Boracchi P, Verderio P and Bevilacqua P (1994) Cell kinetics in human breast cancer: comparison between the prognostic value of the cytofluorimetric S-phase fraction and that of the antibodies to Ki-67 and PCNA antigens detected by immunocytochemistry. *Int J Cancer* **57**: 822–829

Gerdes J, Schwab U, Lemke H and Stein H (1983) Production of a mouse monoclonal antibody reactive with a human nuclear antigen associated with cell proliferation. *Int J Cancer* **31**: 13–20

Gerdes J, Lemke H, Baisch H, Wacker HH, Schwab U and Stein H (1984) Cell cycle analysis of a cell proliferation-associated human nuclear antigen defined by the monoclonal antibody Ki-67. *J Immunol* **133**: 1710–1715

Gerdes J, Li L, Schlueter C, Duchrow M, Wohlenberg C, Gerlach C, Stahmer I, Kloth S, Brandt E and Flad HD (1991) Immunobiochemical and molecular biologic characterization of the cell proliferation-associated nuclear antigen that is defined by monoclonal antibody Ki-67. *Am J Pathol* **138**: 867–873

Gorczyca W, Markiewski M, Kram A, Tuziak T and Domagala W (1995) Immunohistochemical analysis of bcl-2 and p53 expression in breast carcinomas: their correlation with Ki-67 growth fraction. *Virchows Archiv* **426**: 229–233

Graham D, Magee H, Kierce B, Ball R, Dervan P and O'Meara A (1995) Evaluation of Ki-67 reactivity in neuroblastoma using paraffin embedded tissue. *Path Res Pract* **191**: 87–91

Harbeck N, Moniwa N, Busch E, Schmitt M, Jänicke F, Fellbaum C, Höfler H and Graeff H (1991) Durchflußzytometrische DNA-Analyse von reinen Zellkernen aus formalin-fixierten paraffinschnitten beim primären Mammakarzinom: Korrelation mit anderen Prognosefaktoren. *Gynäkol Rundsch* **31**(suppl.2): 299–302

Harbeck N, Yamamoto N, Moniwa N, Schüren E, Ziffer P, Dettmar P, Höfler H, Schmitt M and Graeff H (1994) Flow cytometric DNA analysis in primary breast cancer: Technical pitfalls and clinical applications. In *Elsevier Science: Prospects in Diagnosis and Treatment of Breast Cancer*, Schmitt et al (eds), pp. 63–74

Hedley DW (1989) Flow cytometry using paraffin-embedded tissue: five years on. *Cytometry* **10**: 229–241

Hedley DW, Friedlander ML, Taylor IW, Rugg CA and Musgrove EA (1983) Method for analysis of cellular DNA content of paraffin-embedded pathological material using flow cytometry. *J Histochem Cytochem* **31**: 1333–1335

Hedley DW, Friedlander ML and Taylor IW (1985) Application of DNA flow cytometry to paraffin-embedded archival material for the study of aneuploidy and its clinical significance. *Cytometry* **6**: 327–333

Hedley DW, Rugg CA and Gelber RD (1987) Association of DNA index and S-phase fraction with prognosis of node positive early breast cancer. *Cancer Res* **47**: 4729–4735

Isola JJ, Helin HJ, Helle MJ and Kallioniemi OP (1990) Evaluation of cell proliferation in breast carcinoma. Comparison of Ki-67 immunohistochemical study, DNA flow cytometric analysis and mitotic count. *Cancer* **65**: 1180–1184

Kallioniemi OP (1988) Comparison of fresh and paraffin-embedded tissue as starting material for DNA flow cytometry and evaluation of intratumor heterogeneity. *Cytometry* **9**: 164–169

 © Cancer Research Campaign 1997

Kallioniemi OP, Hietanen T, Mattila J, Lehtinen M, Lauslahti K and Koivula T (1987) Aneuploid DNA content and high S-phase fraction of tumor cells are related to poor prognosis in patients with primary breast cancer. *Eur J Cancer* **23**: 277–282

Kallioniemi OP, Blanco G, Alavaikko M, Hietanen T, Mattila J, Lauslahti K, Lehtinen M and Koivula T (1988) Improving the prognostic value of DNA flow cytometry in breast cancer by combining DNA Index and S-phase fraction. *Cancer* **62**: 2183–2190

Kamel OW, Franklin WA, Ringus JC and Meyer JS (1989) Thymidine labeling index and Ki-67 growth fraction in lesions of the breast. *Am J Pathol* **134**: 107–113

Lelle RJ, Heidenreich W, Stauch G and Gerdes J (1986) Bestimmung der Wachstumsfraktion bei Mammakarzinomen mit Hilfe des monoklonalen Antikörpers Ki-67. *Tumor Diagnostik und Therapie* **7**: 181–185

Lelle RJ, Heidenreich W, Stauch G and Gerdes J (1987) The correlation of growth fractions with histological grading and lymph node status in human mammary carcinoma. *Cancer* **59**: 83–88

McDivitt RW, Stone KR, Craig RB, Palmer JO, Meyer JS and Bauer WC (1986) A proposed classification of breast cancer based on kinetic information. Derived from a comparison of risk factors in 168 primary operable breast cancers. *Cancer* **57**: 269–276

O'Reilly SM, Camplejohn RS, Barnes DM, Millis RR, Rubens RD and Richards MA (1990) Node-negative breast cancer: prognostic subgroups defined by tumour size and flow cytometry. *J Clin Oncol* **8**: 2040–2046

Ruschoff J, Neumann K, Contractor H, Plate K and Thomas C (1990) Assessment of nucleolarorganizer regions by automatic image analysis in breast cancer: correlation with DNA content, proliferation rate, receptor status and histopathological grading. *J Cancer Res Clin Oncol* **116**: 480–485

Sahin AA, Ro J, Ro JY, Blick MB, El-Naggar AK, Ordonez NG, Fritsche HA, Smith TL, Hortobagyi GN and Ayala AG (1991) Ki-67 immunostaining in node-negative stage I/II breast carcinoma. Significant correlation with prognosis. *Cancer* **68**: 549–557

Sigurdsson H, Baldetorp B, Borg A, Dalberg M, Fernö M, Killander D and Olsson H (1990) Indicators of prognosis in node-negative breast cancer. *N Engl J Med* **322**: 1045–1053

Toikkanen S, Joensuu H and Klemi P (1989) The prognostic significance of nuclear DNA content in invasive breast cancer – a study with long-term follow-up. *Br J Cancer* **60**: 693–700

Verheijen R, Kuijpers HJ, van Driel R, Beck JL, van Dierendorck JH, Brakenhoff GJ and Ramackens FC (1989) Ki-67 detects a nuclear matrix-associated proliferation-related antigen. II. Localisation in mitotic cells and association with chromosomes. *J Cell Sci* **92**: 531–540

Veronese SM, Gambacorta M, Gottardi O, Scanzi F, Ferrari M and Lampertico P (1993) Proliferation index as a prognostic marker in breast cancer. *Cancer* **71**: 3926–3931

Vielh P, Chevillard S, Mosseri V, Donatini B and Magdelenat H (1990) Ki-67 index and S-phase fraction in human breast carcinomas. Comparison and correlations with prognostic factors. *Am J Clin Pathol* **94**: 681–686

Weaver DL, Bagwell CB, Hitchcox SA, Whetstone SD, Baker DR, Herbert DJ and Jones MA (1990) Improved flow cytometric determination of proliferative activity (S-phase fraction) from paraffin-embedded tissue. *Am J Clin Pathol* **94**: 576–584

Weidner N, Moore DH and Vartanian R (1994) Correlation of Ki-67 antigen expression with mitotic figure index and tumor grade in breast carcinomas using the novel 'paraffin'-reactive MIB1 antibody. *Hum Pathol* **25**: 337–342

© Cancer Research Campaign 1997